# Characterization of Citrus-Associated *Alternaria* Species in Mediterranean Areas

**DOI:** 10.1371/journal.pone.0163255

**Published:** 2016-09-16

**Authors:** Francesca Garganese, Leonardo Schena, Ilenia Siciliano, Maria Isabella Prigigallo, Davide Spadaro, Anna De Grassi, Antonio Ippolito, Simona Marianna Sanzani

**Affiliations:** 1 Dipartimento di Scienze del Suolo, della Pianta e degli Alimenti, Università degli Studi Aldo Moro, Bari, Italy; 2 Dipartimento di Agraria, Università Mediterranea, Reggio Calabria, Italy; 3 Centro di Competenza per l'Innovazione in campo agro-ambientale-AGROINNOVA, Università degli Studi di Torino, Grugliasco (TO), Italy; 4 Dipartimento di Scienze Agrarie, Forestali e Alimentari, Università degli Studi di Torino, Grugliasco (TO), Italy; 5 Dipartimento di Bioscienze, Biotecnologie e Biofarmaceutica, Università degli Studi Aldo Moro, Bari, Italy; Universita degli Studi di Pisa, ITALY

## Abstract

*Alternaria* brown spot is one of the most important diseases of tangerines and their hybrids worldwide. Recently, outbreaks in Mediterranean areas related to susceptible cultivars, refocused attention on the disease. Twenty representatives were selected from a collection of 180 isolates of *Alternaria* spp. from citrus leaves and fruit. They were characterized along with reference strains of *Alternaria* spp. Micro- and macroscopic characteristics separated most *Alternaria* isolates into six morphotypes referable to *A*. *alternata* (5) and *A*. *arborescens* (1). Phylogenetic analyses, based on endopolygalacturonase (*endopg*) and internal transcribed spacer (ITS), confirmed this finding. Moreover, a five-gene phylogeny including two anonymous genomics regions (OPA 1–3 and OPA 2–1), and the beta-tubulin gene (*ß-tub*), produced a further clustering of *A*. *alternata* into three clades. This analysis suggested the existence of intra-species molecular variability. Investigated isolates showed different levels of virulence on leaves and fruit. In particular, the pathogenicity on fruit seemed to be correlated with the tissue of isolation and the clade. The toxigenic behavior of *Alternaria* isolates was also investigated, with tenuazonic acid (TeA) being the most abundant mycotoxin (0.2–20 mg/L). Isolates also synthesized the mycotoxins alternariol (AOH), its derivate alternariol monomethyl ether (AME), and altenuene (ALT), although to a lesser extent. AME production significantly varied among the six morphotypes. The expression of *pksJ/pksH*, biosynthetic genes of AOH/AME, was not correlated with actual toxin production, but it was significantly different between the two genotypes and among the four clades. Finally, ten isolates proved to express the biosynthetic genes of ACTT1 phytotoxin, and thus to be included in the *Alternaria* pathotype tangerine. A significant correlation between pathogenicity on leaves and *ACTT1* gene expression was recorded. The latter was significantly dependent on geographical origin. The widespread occurrence of *Alternaria* spp. on citrus fruit and their ability to produce mycotoxins might represent a serious concern for producers and consumers.

## Introduction

The fungal genus *Alternaria* is widely and abundantly disseminated in the environment (atmosphere, soil, seeds, and agricultural produce) [1]. It comprises numerous saprophytic, endophytic, and pathogenic species, causing pre- and postharvest deterioration of cereals, fruit, and vegetables. For example, *Alternaria* brown spot is a highly destructive disease of tangerines and tangerine hybrids of worldwide importance [2]. The disease is prevalent in citrus production areas with a Mediterranean climate, characterized by cool, humid winters and hot, arid summers [3]. In Europe, it has been reported in Spain [4], Italy [5], and Greece [6]. It attacks young leaves, twigs and fruit, causing brown to black lesions surrounded by a yellow halo. Severely infected leaves and fruit may fall, and whole shoots may wilt and die. Therefore, under appropriate environmental conditions, significant losses might occur in terms of both yield and marketability of fruit of susceptible cultivars [2, 7]. *Alternaria* spp. can also cause citrus black rot, which occurs mostly as a core rot [8], as well as leaf spot on rough lemon, and Mancha foliar on Mexican lime. However, these two latter diseases are considered of low economic significance because they are limited to nursery and seed production blocks, or occur in very limited areas [3]. Both black rot and brown spot are caused by several ‘small-spored’ *Alternaria* species [7, 9]. Recently, 35 of them were synonymised as *Alternaria alternata*, meaning that *Alternaria* sect. *Alternaria* consists of only 11 species (*A*. *burnsii*, *A*. *tomato*, *A*. *jacinthicola*, *A*. *iridiaustralis*, *A*. *eichhorniae*, *A*. *betae-kenyensis*, *A*. *gaisen*, *A*. *alstroemeriae*, *A*. *longipes*, *A*. *gossypina*, and *A*. *alternata*) and one species complex (*A*. *arborescens*) [10]. Moreover, the use of *formae specialis* (*f*. *sp*.) to indicate isolates morphologically indistinguishable from *A*. *alternata* but infecting specific hosts has been suggested. In particular, at least 16 different *f*. *sp*. epithets occur in literature, *e*.*g*. *A*. *alternata f*. *sp*. *mali*, or *A*. *alternata f*. *sp*. *citri*.

For classification purposes, the production of the numerous secondary metabolites is also used. They are not only significant in terms of food quality and safety, but also play an important role in disease occurrence [1, 11, 12]. For instance, the host-selective toxins (HSTs), low-molecular-weight compounds with a wide spectrum of structures, determine host range or specificity, since they are toxic only to specific plant species, varieties, or genotypes [13]. As these toxins are required to invade tissues and cause disease, they represent pathogenicity factors [14, 15]. The tangerine pathotype of *A*. *alternata*, causing brown spot, produces HSTs known as ACT-toxins. The structure of ACT-toxins is closely related to those of AK- and AF-toxins, which are HSTs produced by the Japanese pear and strawberry pathotypes of *A*. *alternata*, respectively. Two genes controlling AK-toxin biosynthesis (*AKT1* and *AKT2*) have been cloned [16], and more recently, their two ACT-toxin homologues (*ACTT1* and *ACTT2*), involved in the biosynthesis of the decatrienoic acid moiety [16], have been sequenced [17].

Among secondary metabolites, there are also several mycotoxins, which might pose a risk to human health [11]. The ones occurring most frequently in nature are alternariol (AOH) and its derivate alternariol monomethyl ether (AME), tenuazonic acid (TeA), tentoxin (TEN), altenuene (ALT) and altertoxin I, whereas iso-altenuene and altertoxin II have not been found in crops to date. TeA has cytotoxic and phytotoxic properties, and its LD_50_ value in mice is similar to that of *Fusarium* deoxynivalenol [18]. Altertoxins (ALXs) are considered extremely toxic and stronger mutagens to mice than AOH and AME [19]; moreover, the high genotoxicity of ALX II to mammalian cells has been proven [20, 21]. AOH is formed along the polyketide route, which is a common pathway for the formation of many secondary fungal metabolites [22, 23]. Biosynthetic routes for AOH have been studied extensively [24, 25]. More recently, in a draft genome sequence of *A*. *alternata*, 10 putative polyketide synthases (pks)-encoding genes have been identified [22]. Among them, *pksJ* proved to be required for biosynthesis, whereas, *pksH* downregulation proved to affect *pksJ* expression, thus having an indirect effect on AOH production [22].

In the present study, specific investigations were made to gain insight into recent *Alternaria* disease outbreaks in the Mediterranean basin. The aim of the study was to characterise the main species involved, also in relation to their ability to produce mycotoxins, in order to draw up appropriate control strategies.

## Materials and Methods

### Ethics Statement

This study did not involve endangered or protected species. No ethical permits were required for this work, which involved no experimentation on animals or human samples.

### Isolates used in the study

Twenty representative isolates were selected from the *Alternaria* spp. collection of the Department of Soil, Plant and Food Sciences, University of Bari Aldo Moro, Italy. In particular, they were selected among citrus isolates and according to tissue (leaf/fruit) and geographical area of isolation, *i*.*e*. Italy (GPS coordinates 40°20'39.2"N and 16°43'18.5"E) and Spain (GPS coordinates 39°56'42.993"N and 0° 3′ 38.389″ W) (Table 1).

**Table 1:** Selected *Alternaria* spp. isolates used in the study and their origins.

Isolate	Location	Tissue	Accession numbers				
			ITS	ß-tub	endoPG	OPA1-3	OPA2-1
**A22**	Spain	Fruit	KU933192	KU933152	KU933172	KU933212	KU933232
**A23**	Spain	Fruit	KU933193	KU933153	KU933173	KU933213	KU933233
**A24**	Spain	Fruit	KU933194	KU933154	KU933174	KU933214	KU933234
**A25**	Spain	Fruit	KU933195	KU933155	KU933175	KU933215	KU933235
**A26**	Spain	Fruit	KU933196	KU933156	KU933176	KU933216	KU933236
**A27**	Spain	Leaf	KU933197	KU933157	KU933177	KU933217	KU933237
**A28**	Spain	Leaf	KU933198	KU933158	KU933178	KU933218	KU933238
**A29**	Spain	Leaf	KU933199	KU933159	KU933179	KU933219	KU933239
**A30**	Spain	Leaf	KU933200	KU933160	KU933180	KU933220	KU933240
**A31**	Spain	Leaf	KU933201	KU933161	KU933181	KU933221	KU933241
**A41**	Italy	Leaf	KU933202	KU933162	KU933182	KU933222	KU933242
**A42**	Italy	Leaf	KU933203	KU933163	KU933183	KU933223	KU933243
**A43**	Italy	Leaf	KU933204	KU933164	KU933184	KU933224	KU933244
**A44**	Italy	Leaf	KU933205	KU933165	KU933185	KU933225	KU933245
**A45**	Italy	Leaf	KU933206	KU933166	KU933186	KU933226	KU933246
**A63**	Italy	Fruit	KU933207	KU933167	KU933187	KU933227	KU933247
**A64**	Italy	Fruit	KU933208	KU933168	KU933188	KU933228	KU933248
**A65**	Italy	Fruit	KU933209	KU933169	KU933189	KU933229	KU933249
**A66**	Italy	Fruit	KU933210	KU933170	KU933190	KU933230	KU933250
**A67**	Italy	Fruit	KU933211	KU933171	KU933191	KU933231	KU933251

### Morphological characterization

Selected isolates were characterized along with reference strains of *A*. *alternata* (CBS 107.27, CBS 112252, CBS 112249, CBS 102600, CBS 102595) and *A*. *arborescens* (CBS 109730) from CBS-KNAW (Utrecht, The Netherlands). All 20 isolates were grown on Potato Dextrose Agar (PDA, Oxoid, Milan, Italy), Malt Extract Agar (MEA, Oxoid), and Potato Carrot Agar (PCA, prepared according to CBS-KNAW, http://www.cbs.knaw.nl/Collections/BioloMICS.aspx?Table=Growth%20media).

To determine colony morphology, plates were incubated for 10 days at 22±1°C in the dark. The macroscopic characteristics (colour, margin, diameter, and texture) were analysed as reported by Pryor and Michailides [26].

To study the microscopic characteristics, after 5 days of incubation in the dark, a rectangular block (10×20 mm) of agar and mycelium was removed aseptically within the expanding margin of the colony, exposed to daylight and returned to incubation surface. On day 10, it was put on a slide and gently pressed with a glass slip. For each isolate, observations were made at ×40 magnification and sub-stage illumination to highlight the sporulation apparatus features. The sporulation characteristics were compared with those reported in the *Alternaria* identification manual [27] and by Pryor and Michailides [26].

### Molecular characterization

A multilocus approach was adopted to characterize and determine the phylogenetic collocation of the 20 selected isolates from citrus. Portions of five *Alternaria* barcoding genes/regions, *i*.*e*. internal transcribed spacer (ITS), endopolygalacturonase (*endopg*), two SCAR markers (OPA 1–3 and OPA 2–1), and beta-tubulin *(ß-tub*), were sequenced. Fungal isolates were grown for 7 days on PDA at 24±1°C in the dark, and mycelia were collected and stored at -20°C until use. Genomic DNA was extracted from 100 mg of mycelium as reported by Sanzani et al. [28], and diluted to 50 ng/μl in sterile ultra-pure water. PCR was carried out in a 50 μl reaction mixture containing 50 ng of DNA, 10 μM of each primer, and 5U of Taq DNA Polymerase (EmeraldAmp PCR master mix, Takara, Clontech, USA). PCR was carried out in a thermal cycler (MyCycler, BioRad, Hercules, CA, USA), using reported cycling conditions [8, 29, 30, 31]. All primer pairs were synthesized by Sigma-Aldrich (Milan, Italy).

Amplicons were resolved in 1% agarose gels in TAE buffer (1×) and visualized by UV illumination. DNA was recovered from agarose by Isolate II Genomic DNA kit (Bioline, London, UK) according to manufacturer recommendations. Purified products were sequenced with both forward and reverse primers by Macrogen Europe (Amsterdam, The Netherlands). CHROMASPRO v. 1.5 software (http://www.technelysium.com.au/) was used to evaluate sequence reliability, and the consensus sequences were deposited in Genbank (Table 1). Unreliable sequences containing doubtful bases were re-sequenced.

For the molecular identification of isolates, ITS and *endopg* sequences obtained in the present study, and validated sequences representative of all species identified within *Alternaria* Sect. *Alternaria* were phylogenetically analysed to determine the taxonomic status of isolates in the light of recent molecular criteria for classification [10]. Before analyses, the complete panel of reference sequences was analysed utilizing the ElimDupes software (http://hcv.lanl.gov/content/sequence/ELIMDUPES/elimdupes.html) to delete multiple identical sequences. Identical reference sequences were included in the panel when representative of different *Alternaria* species [10]. Sequences were aligned using MUSCLE and introduced to MEGA6 for phylogenetic analysis with the Maximum Likelihood method using the Tamura-Nei model [32]. Analyses were performed with 500 bootstrap replications.

In order to maximize the effectiveness of the investigation into the genetic diversity within isolates from citrus obtained in the present study, the phylogenetic analysis was repeated using a combined data set of all sequenced markers (ITS, *endopg*, OPA 1–3, OPA 2–1, and *ß-tub*).

### Pathogenicity tests

The ability of isolates to cause brown spot was assessed *in vivo* on freshly harvested citrus fruit and leaves (*Citrus reticulata* cv. Fortune), free of defects or injuries, and uniform in size, colour and ripeness, collected in Metaponto (Italy, GPS coordinates 40°23'17.611''N, 16°47'48.799''E). The selected citrus fruit and leaves were randomized, surface-sterilized with 1% sodium hypochlorite, and extensively washed under running tap water. After air-drying, they were wounded and kept at room temperature for 30 min; each fruit was wounded with a sterile nail (3×3 mm) in two equidistant points on the equatorial surface, leaves were wounded by pricking the abaxial side with 3 entomological pins mounted on a cork. For each isolate, a drop (10 μl) of conidial suspension (10^5^ conidia/ml), prepared by flooding plates with 5 ml sterile 0.02% Tween20 solution and gently rubbing the colony surface with a sterile spatula, was applied into the wounds of 24 fruit and 18 leaves that were then randomly divided into three replicates. Samples were incubated at 24±1°C and high relative humidity for 10 days. The disease incidence (infected wounds, %) and severity (lesion diameter, mm) were recorded. The assay was repeated twice.

### Expression analyses

The selected isolates (in triplicate) were grown in 30 ml of Potato Dextrose Broth (PDB, Oxoid) on a rotary shaker (150 rpm), in the dark at 24±1°C. After 10 days, the mycelium was harvested, lyophilized and kept at -80°C. The culture broth was stored at -20°C until mycotoxin analysis.

Total RNA was extracted from 100 mg of mycelium using Plant RNA/DNA Purification Kit (Norgen Biotek, ON, Canada) and purified by treatment with DNaseI following manufacturer’s instructions. The total RNA obtained was visualized on a 1.5% (w/v) agarose gel and quantified by a NanoDrop Spectrophotometer (Thermo Scientific, Wilmington, USA). Then, it was serially diluted in the range 0.1–1000 ng, and the dilutions were reverse-transcribed using an iScript cDNA synthesis kit (BioRad). Primers obtained from regions of genes *ACTT1* and *ACTT2* (phytotoxins), and *pksJ* and *pksH* (mycotoxins) were used in real time quantitative PCR (qPCR) reactions (Table 2).

**Table 2:** Primer used in gene expression studies. Sequences, linear equations, determination coefficients (R^2^), and reaction efficiencies (RE, %), obtained by plotting serially diluted RNA concentrations (log scale) and corresponding Ct values experimentally determined in real time PCR reactions, for genes *pksJ*, *pksH*, *ACTT1*, and *ACTT2*.

Gene	Primer name	Sequence (5’-3’)	Linear equation	R^2^	RE (%)	Source
*pksJ*	• pksJ_RT_fw_N	• GTCCCAAATTCCTACCCTCAC	y = -3.25x + 33.10	0.98	102.9	[22]
	• pksJ_RT_rv_N	• GATAGCCATCGAAAGCATTCCC				
*pksH*	• pksH_RT_fw_N	• GTCAACCCTCTCACACCAAC	y = -3.59x + 37.45	0.99	90.0	[22]
	• pksH_RT_rv_N	• GACGCATCGCTTCAATAGCC				
*ACTT1*	• HACT1F	• ATGCGCGAGATTTTCTGACC	y = -3.50x + 36.18	0.99	92.9	This study
	• HACT1R	• CTGTCTCCCCGGTACAAAGT				
*ACTT2*	• HACT2F	• TGACATTACGACGTAGGACGC	y = -3.24x + 31.52	0.96	103.3	This study
	• HACT2R	• GCTCCTGATATCGTCCTGTGA				

Standard curves were generated by plotting Cycle threshold (Ct) values (y-axis) against logs of total RNA (x-axis). Reaction efficiency, precision, and dynamic range were calculated [33]. The cDNA used for the expression reactions was synthesised from 500 ng total RNA. The amplification mixture (10 μl) contained 1 μl of template, 5 μl of SensiFAST SYBR & Fluorescein Mix (Bioline), 3.2 μl of ultra-pure H_2_O and 0.4 μl of each specific primer (10 μM). In negative control samples, cDNA was replaced with sterile water or non-reverse transcribed RNA to exclude possible cross-contamination and the presence of genomic DNA, respectively. Amplifications were performed in 96-well reaction plates using an iCycler iQ thermal cycler (BioRad). The amplification program consisted of 1 cycle of 95°C for 2 min, followed by 40 cycles of 95°C for 5 s, 62 (*pksJ-pksH*)/60 (*ACTT1-ACTT2*) °C for 10 s and 72°C for 10 s. Subsequently, a melting curve analysis was done to determine the reaction specificity by incubating the samples at 95°C for 2 min, annealing at 65°C for 5 s, followed by heating them slowly at 0.5°C/sec up to 95°C, while continuously monitoring the fluorescence signal. Moreover, an aliquot (5 μl) of product was run on a 1% agarose gel. Ct was automatically generated by the iCycler associate software (Real Time Detection System Software, version 3.0). The absolute expression was calculated by interpolation from the calibration curve.

When there was no expression, specific PCR reactions were conducted to assess the presence of *ACTT1* and *ACTT2* genes. The primers were SACT1F 5’-CACAGGCTATCTTCACATGCA-3’/SACT1R 5’-GCATTGCTTTATCTTCCACGT-3’ and SACT2F 5’-CCTTCTTGTGTGCCGGAAAA-3’/SACT2R 5’-TGCTACAAGTTACGAGGCCA-3’. The reaction mixture and conditions were those reported for EmeraldAmp PCR master mix (Takara), at an annealing temperature of 60°C.

### Mycotoxin analyses

A method for the simultaneous detection of the main five *Alternaria* toxins (ATs) was applied [34]. A liquid chromatography–triple quadrupole mass spectrometer (Applied Biosystems, Foster City, CA, USA) equipped with atmospheric pressure chemical ionisation (APCI) was used. The culture broth was filtered through filter paper (Whatman No 4, GE Healthcare, Milan, Italy) and re-filtered using a sterile 45μ syringe filter (Bibby Scientific Limited, Stone, Staffordshire, UK). Filtrates were extracted as reported by Prelle et al. [34] and analysed as mentioned above. The entire experiment was repeated twice.

## Statistical analysis

All statistical analysis were conducted in R (https://www.r-project.org/). Ten data samples in replicates, *i*.*e*. pathogenicity on leaf, pathogenicity on fruit, four toxins, and *pksH*, *pksJ*, *ACTT1* and *ACTT2* expression, were independently tested for normality using the Shapiro-Wilk (SW) test and Q-Q plot inspection. Given that samples did not come from normally distributed populations (SW test p < 0.05), nonparametric tests were chosen for downstream analyses. Each sample was divided into distinct classes, *i*.*e*. two origins, two tissues, six morphotypes, two genotypes and four clades. The two-ways Wilcoxon (W) test was applied for comparing samples from two classes and the Kruskal-Wallis (KW) test for three or more classes. A Heatmap was built on the full data matrix using default parameters in ClustVis (http://biit.cs.ut.ee/clustvis/).

## Results

### Morphological characterization of isolates

All 20 isolates were grouped according to macro- and microscopic features on PDA, MEA, and PCA, along with reference strains of *A*. *alternata* and *A*. *arborescens* from CBS-KNAW. A higher variability in colony morphology was observed on PDA, as compared to PCA and MEA (data not shown), allowing the differentiation of six morphotypes. In particular, twelve isolates (A22, A23, A25, A27, A28, A29, A31, A45, A63, A64, A65, A67) showed colonies that were flat, woolly and with colours ranging from brown to black, and an average diameter of 65 mm. They produced dark brown conidia arranged in branched chains. Conidia appeared oval-ellipsoidal with 3–5 transverse septa. These features matched those of *A*. *alternata* reference strain CBS 112249 (morphotype 1).

Two isolates (A24, A43) showed colonies varying from greenish-grey to brown, characterized by a lower growth rate (average diameter 45 mm after 7 days on PDA). Conidia appeared oval or ellipsoidal with 1–4 transverse septa and 1–2 longitudinal septa. Conidia were borne by long primary conidiophores, occasionally presenting sub-terminal branches. These characteristics matched those of the reference strain for *A*. *arborescens* CBS 109730 (morphotype 2).

Three isolates (A26, A30, A41) showed greenish colonies with white margins. Conidia appeared elongated with a long tapered beak. The characteristic sporulation pattern matched that of the reference strain for *A*. *alternata* (*ex tenuissima*) CBS 112252 (morphotype 3). One isolate (A42) exhibited a colony pale brown, flat, granulated with undulating edges. Conidia appeared long and ellipsoidal with 1–3 transverse septa. The sporulation pattern resembled that of *A*. *alternata* (*ex limoniasperae*) CBS 102595 (morphotype 4). One isolate (A44) exhibited a sporulation pattern resembling that of the reference strain of *A*. *alternata* (*ex citri*) CBS 107.27, with elliptical and subglobose conidia (morphotype 5); finally, isolate A66 exhibited wide and long conidia, with a sporulation pattern resembling that of *A*. *alternata* (*ex toxicogenica*) CBS 102600 (morphotype 6).

### Molecular characterization

**Fig 1:**
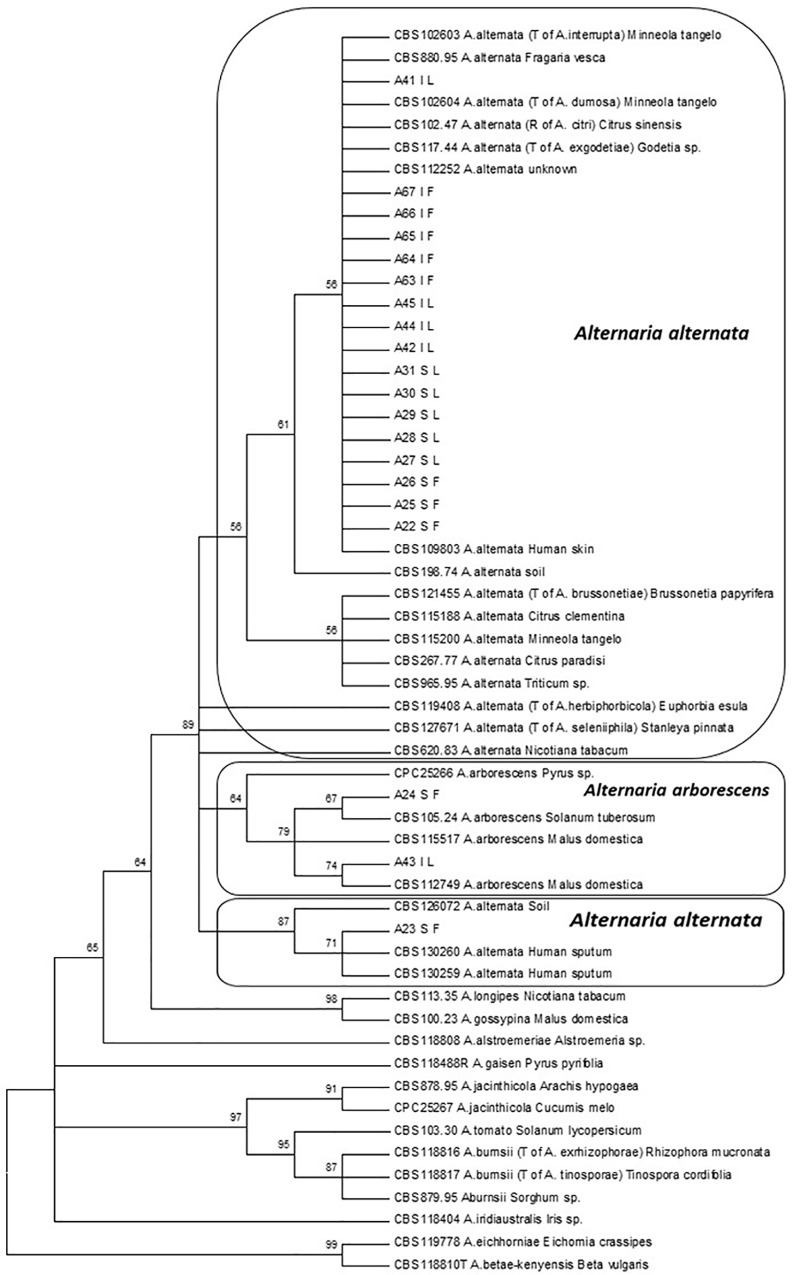
Phylogenetic tree based on the internal transcribed spacer (ITS) and endopolygalacturonase (*endopg*) sequences of 20 isolates of *Alternaria* spp. They are reported according to geographical origin and tissue of isolation: I (Italy), L (leaf), S (Spain), and F (fruit). For the analysis, 156 *Alternaria* reference strains were initially included, then reduced to 24 after exclusion of duplicates. Numbers on nodes represent the maximum likelihood bootstrap percentages. Branch lengths are proportional to the numbers of nucleotide substitutions and are measured using the bar scale (0.002). Species names in parentheses represent synonymised species names. Ex-type strains are indicated with a T and representative strains, used to describe the species based on morphology in Simmons (2007), with an R.

A two-gene phylogeny, including ITS and *endopg* sequences, of isolates selected in the present study and reference CBS strains [10] was performed to identify isolates (Fig 1). According to this analysis, 18 out of 20 isolates were associated to *A*. *alternata*. They were almost equally distributed as regards the two geographical areas (Spain or Italy) and tissue of isolation (leaf or fruit), and clustered with several reference isolates of *A*. *alternata*, including the strain CBS 112252 (Fig 1). Finally, two isolates namely A24 (Spain, fruit) and A43 (Italy, leaf) grouped within the species complex of *A*. *arborescens* along with strains CBS 105.24 from *Solanum tuberosum* and CBS 112749 from *Malus domestica*.

**Fig 2:**
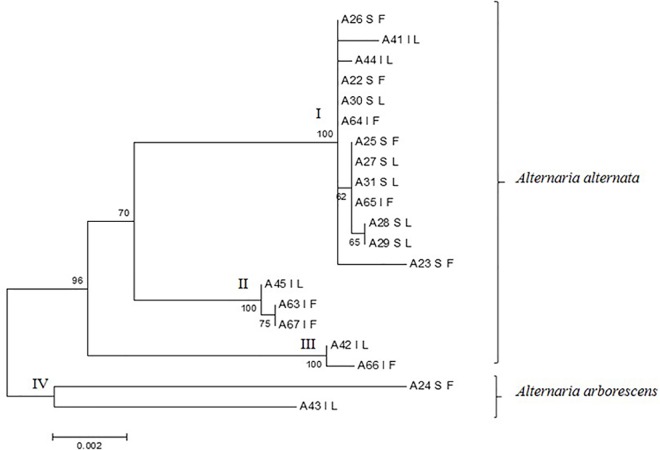
Phylogenetic tree based on the internal transcribed spacer (ITS), endopolygalacturonase (*endopg*), beta tubulin (*ß-tub*), and SCAR markers OPA1-3 and OPA2-1 sequences of 20 isolates of *Alternaria* spp. They are reported according to geographical origin and tissue of isolation: I (Italy), L (leaf), S (Spain), and F (fruit). Numbers on nodes represent the maximum likelihood bootstrap percentages. Branch lengths are proportional to the numbers of nucleotide substitutions and are measured using the bar scale (0.002).

The phylogenetic analysis of isolates using all sequenced genes (ITS, *endopg*, *ß-tub*, OPA1-3 and OPA2-1) made it possible to achieve a more accurate discrimination and to identify four main clades (Fig 2). In particular, three clades, represented by 13, 3 and 2 isolates, respectively, were identified in *A*. *alternata* (Fig 2). One additional clade, represented by isolates A24 and A43, corresponded to the *A*. *arborescens* species complex. A partial matching was found between molecular clades and morphotypes. In particular, isolates of clade II belonged to morphotype 1 (*alternata*), which was also the prevalent morphotype in clade I. The latter included also morphotypes 3 and 5 (*ex-tenuissima* and *ex-citri*). Isolates of the *A*. *arborescens* species complex (clade IV) were characterized by the same morphotype (2). Finally, clade III included morphotypes 4 (*ex-limoniasperae*) and 6 (*ex-toxicogenica*).

### Pathogenicity Tests

**Fig 3:**
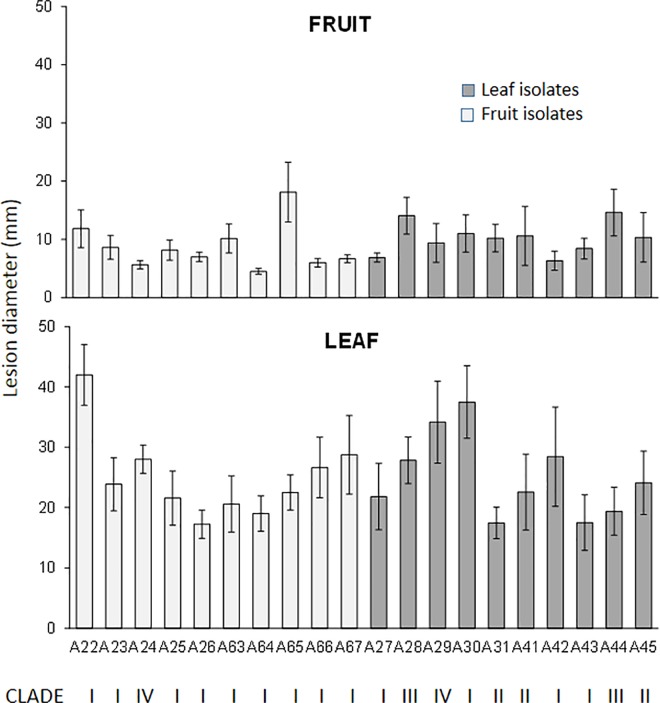
Virulence (lesion diameter, mm) of 20 *Alternaria* spp. isolates on detached leaf and fruit of *Citrus reticulata* cv. Fortune. Samples were wounded, inoculated, and incubated at 24±1°C in the dark, 90–95% RH, for 10 days. For each isolate, data are the average of 18 (leaves)/24 (fruit) wounds ± standard error of the mean (SEM). Leaf isolates are represented in light grey, whereas fruit isolated in dark grey. For each isolate, the related clade is indicated.

Pathogenicity assays foresaw the evaluation of disease incidence and severity up to 10 days from inoculation on both fruit and leaves. All tested isolates caused necrosis around the inoculation point, expanding into leaf and fruit tissues (Fig 3). Similar necrotic symptoms were observed, regardless of origin and genetic groupings. The lesions developed initially as rapidly enlarging, almost circular, brown, water-soaked spots that quickly turned into dark brown, necrotic spots surrounded by yellowish margins. Lesions on leaves, ranging from 17 to 42 mm, were larger than those on fruit, ranging from 5 to 18 mm (W test p < 0.001). The pathogenicity on fruit was slightly higher in leaf-deriving isolates compared with fruit-deriving isolates (W test p < 0.05), and it also differed among the isolates from the four clades (KW test p < 0.005); instead, the pathogenicity on leaves was not found to differ in terms of geographical origin, tissue of isolation, morphotype, genotype or clade.

### Phytotoxin and mycotoxin biosynthetic gene expression

**Fig 4:**
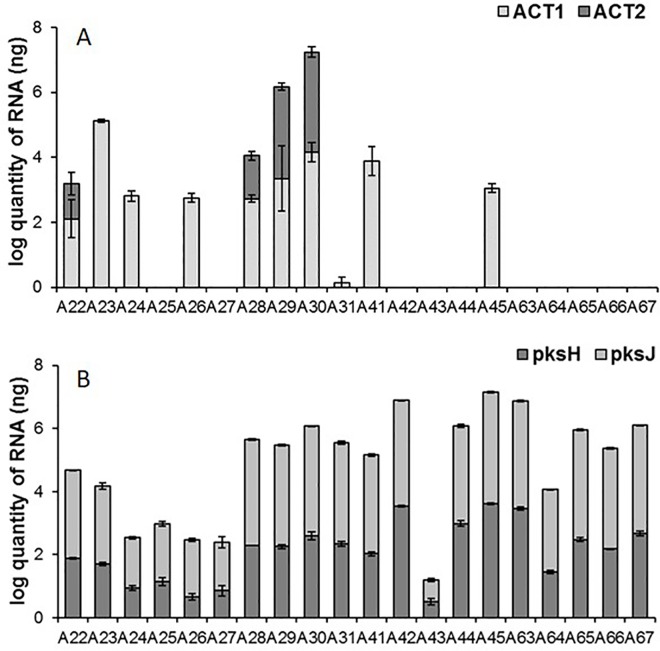
Gene expression analyses. Expression of *ACTT1* and *ACTT2* genes of the biosynthesis of phytotoxins ACTT1 and ACTT2 (A), and *pksH* and *pksJ* genes of the biosynthesis of mycotoxins alternariol and alternariol monomethyletere (B) in the 20 *Alternaria* isolates. They were grown in the dark at 24±1°C for 10 days. For each isolate, data are the average of three replicates ± standard error of the mean (SEM).

The expression analysis of the genes *ACTT1* and *ACTT2* is reported in Fig 4A. Primer pairs were designed upon gene sequences available in Genbank and for each one standard curves were drawn up (Table 2). An absolute quantification of gene expression was performed by interpolation from the standard curves. Specific qPCR reactions were conducted for the 20 selected isolates. After 10 days of incubation at 24°C, *ACTT1* was expressed at detectable levels only in 10 isolates (*i*.*e*. A22, A23, A24, A26 A28, A29, A30, A31, A41, A45) and the absolute quantity of total RNA was 1–4 log (ng). Similarly, *ACTT2* was expressed in 4 out of the 20 tested isolates, namely A22, A28, A29 and A30, and the absolute quantity of total RNA was 1–6 log (ng). PCR reactions were conducted to confirm the absence of coding genes (data not shown).

Moreover, specific qPCR reactions were performed using primer pairs for *pksJ* and *pksH*, whose standard curves were drawn up for absolute quantification (Table 2). Results showed that, after 10 days of incubation at 24±1°C, there was a 1–3.5 log of transcript (ng) for *pksH*, and a 1.5–3.5 log for *pksJ* (Fig 4B).

A positive correlation (linear regression R^2^ = 0.70, p < 0.05) was found between *ACTT1* expression level and pathogenicity on the leaves. Moreover, *ACTT1* expression was 3-fold higher in isolates from Spain compared with those from Italy (W test p < 0.05); differences were also found among the six morphotypes (KW test p < 0.05). *ACTT2* expression was significantly related to isolate origin (p < 0.005). Because of ACT toxins production, isolates A22, A23, A24, A26, A28, A29, A30, A31, A41, A45 could be defined as *A*. *alternata f*. *sp*. *citri* pathotype tangerine.

The expression of the two *pksJ* and *pksH* genes was linearly correlated (linear regression R^2^ = 0.78, p < 0.0001), and A43 (*A*. *arborescens*) was the isolate in which the expression of the two genes was lower. The expression of each gene was higher in Italian isolates compared to Spanish isolates (W test p < 0.05), and it significantly varied among different clades (KW test p < 0.0005).

### Mycotoxin analyses

The toxigenic potential of the selected *Alternaria* spp. isolates was investigated *in vitro* on liquid cultures (Table 3). In particular, they were assayed for the production of TeA, AOH, AME, ALT, and TEN. All tested isolates proved to produce TeA, AOH, AME, and ALT, whereas TEN was not detected in any of the samples. Overall, TeA was the most abundant toxin, produced in the range of 0.2–20 mg/l, with A23 (*A*. *alternata*) and A43 (*A*. *arborescens*) being the highest and lowest producers, respectively. TeA was 1.6-fold more abundant in isolates from fruit compared to isolates from leaves (W test p < 0.05). A consistent production of ALT was also detected, in the range of 0.08–8 mg/l. ALT levels were 3-fold higher in Italian isolates compared with Spanish ones (W test p < 0.005), 2-fold higher in fruit isolates than in leaf ones (W test p < 0.001), and also varied among distinct clades (KW test p < 0.05). AOH and its derivate product AME were detected in all samples in the range of 30–200 and 20–300 μg/l, respectively. The highest AOH producers were A23 and A44; AME production differed significantly among distinct morphotypes (KW test p < 0.005) and clades (KW test p < 0.05).

**Table 3:** HPLC-MS/MS analyses of mycotoxins. Production (μg/l) of alternariol (AOH), alternariol monomethyletere (AME), altenuene (ALT) and tenuazonic acid (TeA) by 20 *Alternaria* isolates *in vitro*. They were grown in the dark at 24±1°C for 10 days. For each isolate, data are the average of three replicates ± standard error of the mean (SEM).

	AOH	AME	ALT	TeA
**A22**	41 ± 7	25 ± 7	4093 ± 650	4076 ± 246
**A23**	178 ± 47	105 ± 63	1495 ± 146	18559 ± 27
**A24**	105 ± 14	22 ± 3	33 ± 24	11029 ± 756
**A25**	103 ± 28	219 ± 60	1115 ± 215	7544 ± 92
**A26**	27 ± 11	132 ± 30	974 ± 80	1082 ± 15
**A27**	94 ± 32	104 ± 6	351 ± 52	8979 ± 62
**A28**	60 ± 9	261 ± 22	162 ± 16	4680 ± 648
**A29**	24 ± 3	134 ± 58	979 ± 563	4181 ± 564
**A30**	51 ± 7	45 ± 26	615 ± 190	4060 ± 357
**A31**	31 ± 0	47 ± 20	99 ± 65	5729 ± 867
**A41**	33 ± 18	37 ± 1	602 ± 117	2214 ± 627
**A42**	13 ± 1	21 ± 11	1881 ± 139	9570 ± 1355
**A43**	61 ± 29	29 ± 6	667 ± 499	62 ± 8
**A44**	132 ± 36	270 ± 36	2145 ± 90	9306 ± 35
**A45**	54 ± 3	162 ± 21	1701 ± 828	1587 ± 170
**A63**	68 ± 20	71 ± 29	4240 ± 190	6901 ± 1440
**A64**	54 ± 25	28 ± 1	7886 ± 651	4442 ± 1431
**A65**	34 ± 8	142 ± 16	1674 ± 228	10033 ± 1801
**A66**	46 ± 29	114 ± 37	1968 ± 1083	8613 ± 1573
**A67**	43 ± 23	186 ± 57	6790 ± 1100	11635 ± 1247

**Fig 5:**
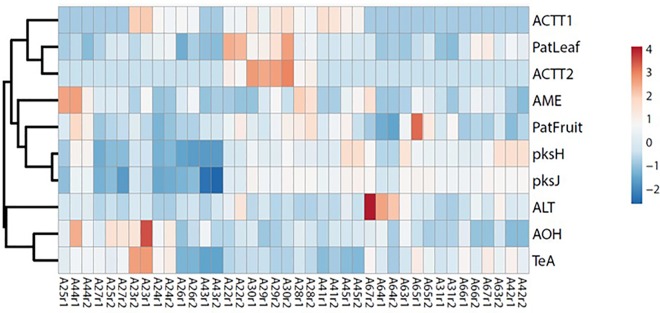
Heatmap representation of the full data set of isolates and variables. Pathogenicity on fruit and leaf are indicated as PatFruit and PatLeaf, respectively. Measure replicates are indicated as rep1 and rep2 for each isolate. The shades of blue and red indicate the gradients eventually present. For color image, the reader may refer to the web version of this article.

The heatmap analysis clustered the full data set according to isolates and variables (Fig 5). The cluster of variables grouped *ACTT* gene expression with pathogenicity on leaves, whereas the expression of both *pks* genes grouped with pathogenicity on fruit and AME. The other mycotoxins constituted an out-group with a poorly uniform pattern of values.

## Discussion

This work encompasses studies on the diversity of *Alternaria* isolates recovered from citrus plants (fruit and leaves) affected by brown spot in Mediterranean countries relevant to citrus production. They were conducted using morphological, pathogenic, toxigenic, and molecular approaches, since only a polyphasic approach could be useful in the characterization of the genus *Alternaria* [35, 36]. Concerning morphological characterization, interesting results were observed when isolates were grown on PDA. Indeed, as also reported by Pryor and Michailides [26], on PDA *Alternaria* produces conidiophores that are much less dense as compared to other cultural media, and thus a better observation of typical structures and associated conidial chains is possible. Moreover, the use of ready-to-use commercially available media guarantees greater batch-to-batch uniformity as compared to locally prepared non-commercial formulations, although the firmness and reproducibility of morphological descriptions would surely benefit greatly from the development of a specific medium suitable for the sporulation of *Alternaria* spp. [26]. Although we are aware of studies concerning the influence of light/dark cycle on toxin production [37], they refer to another growth medium (mCDB) than the one used in the present investigation (PDA/PDB). Pruß and co-workers [37] found that AOH and AME were produced in considerable amounts even in the dark. Moreover, in other similar papers, *Alternaria* strains were incubated in the dark prior to mycotoxin analysis [38, 39, 40]. Since the growth conditions of our isolates were the same in all assays, morphology, pathogenicity, and toxigenicity results could be compared.

Almost all isolates included in the study proved to match with *A*. *alternata*, which is the most widespread species across plants, seasons, and geographical regions, including host-specific pathogenic strains, as well as opportunistic and saprophytic forms causing the spoilage of freshly harvested crops [41]. Within this species, in the present study five morphotypes were identified according to colony and sporulation apparatus features, which correlated well with the CBS reference strains included for comparative purposes, although there were minor differences among isolates within each morphotype. In particular, morphotype 1 (*A*. *alternata*) was more abundant than the others, independently from geographical or tissue origin. However, a higher number of morphotypes was recorded among Italian isolates.

On the basis of the work by Woundenberg et al. [10], phylogenetic analyses were initially conducted by combining ITS region and *endopg* gene. It made it possible to cluster 18 out of 20 isolates with *A*. *alternata* CBS references, and the remaining two isolates with *A*. *arborescens* CBS references, thus confirming that isolates of the *arborescens* species complex are molecularly distinct from isolates of the *alternata* species-group. However, in the present study we observed that when further genes/regions (*β-tub*, OPA 1–3, OPA 2–1) were included in the analysis, a higher variability within *A*. *alternata* species was found. In particular, three clades were obtained, which, however, did not perfectly overlap with the morphotype grouping. Generally, the highest variability was observed in OPA1-3 region sequences, as already reported by Stewart et al. [42]. These results should be taken into account in the future studies on the characterization of *Alternaria*.

All of the investigated isolates were pathogenic to both leaves and fruit, although isolates proved to be more virulent on leaves. On the contrary, Huang et al. [41] and Peever et al. [7] found 15 and 8%, respectively, of non-pathogenic strains under inoculum conditions in their collections. A relation was found between pathogenicity on fruit and clades, with clade I the most pathogenic. Moreover, leaf isolates were slightly more pathogenic on fruit than the other isolates. This finding is not surprising, since leaf isolates expressed more phytotoxin genes that are known to be involved in *Alternaria* disease outbreak and progression. In particular, the tangerine pathotype produces ACTT1 and ACTT2, with toxin 1 being the most abundant [43]. A correlation was found between pathogenicity on leaves and *ACTT1* expression, which was to be expected since ACTT1 is toxic to both citrus and pear (causing venal necrosis and a rapid increase in electrolyte loss from susceptible leaves), whereas ACTT2, the 5″-deoxy derivative of ACTT1, is highly toxic to pear and slightly toxic to citrus [44]. In our study, 10 isolates (eight Spanish and two Italian) expressed *ACTT1* gene and four of them (A22, A28, A29, A30, all Spanish) also expressed *ACTT2* gene. Morphotype 1 was the most represented. We are attempting to quantify the actual phytotoxin presence in inoculated tissues; however, the analysis is made difficult on account of the lack of a true standard.

Despite the well-known role of ACTs as pathogenicity factors, all of the tested isolates were able to cause brown spot, even in the absence of *ACTT1/ACTT2* expression. These isolates proved to produce mycotoxins (TeA, ALT, AOH, and AME), whose role in several host-pathogen interactions has been analysed. They have been reported to be involved in the disease appearance/progression in *Penicillium expansum*-apple [23], *Fusarium graminearum*-cereals [45], but also in *A*. *alternata*-tomato [46] interactions. Therefore, mycotoxins might have played a role in the pathogenicity of the selected isolates. Indeed, as evidenced also by heatmap analysis, the expression of *pksJ* and *pksH* involved in AOH/AME biosynthesis was related to pathogenicity on fruit. Moreover, *pks* expression in Italian isolates (only two expressing *ACT* genes) was higher than in Spanish ones. Our feeling is that mycotoxins effect could sum up to phytotoxin in disease outcome, meaning that in the absence of phytotoxins, a certain degree of virulence is in any case guaranteed. However, this hypothesis needs to be validated by specific assays. In addition, the *pks* expression seemed to be related to the clade.

Concerning mycotoxin production, TeA was the most abundant one, as already reported for tomatoes by Van de Perre et al. [47]. An inter-clade variability was present for AME, as well as a difference among morphotypes for both AOH and AME. ALT was more abundant in Italian isolates coming from fruit. However, as also reported by Andersen et al. [48], chemotaxonomy alone cannot be considered a reliable tool for *Alternaria* identification. Considering the ability of the isolates to produce mycotoxins in considerable amounts, and the increasing concern of consumers and distributors as to their presence in both fresh and processed products, in the near future there will be a need for regulatory limits and monitoring strategies not only for crops, including citrus, but also processed food products (juices, purees, etc.). In fact, a dietary exposure assessment, performed for TeA with Belgian consumption data, revealed a mean value (4230 ng/kg bw/day) higher than the threshold value of toxicological concern (TTC) of 1500 ng/kg bw/day set by EFSA [11].

## Conclusions

In conclusion, *Alternaria*-citrus represents an interesting pathosystem, since the fungus is a common saprophyte on citrus leaves and fruit in the grove, but it is able to turn into a pathogen under suitable conditions. In our study, most of the isolates belonged to *A*. *alternata f*. *sp*. *citri* pathotype tangerine morphotype *alternata* (S1 Table). Overall, a variability in morphotypes was observed among isolates from Italy. Pathogenicity was stronger on leaves than on fruit, and appeared to be related not only to ACTs, more abundantly produced on leaves, but also to other pathogenicity factors such as mycotoxins, produced by all isolates. This finding seems particularly true as far as pathogenicity on fruit is concerned. The different parameters analysed were not often related. It must be remembered that mycotoxin production depends on the geographical location where the plant is grown and harvested as well as on climate, the type of plant, and cultivar affected [40]. Similarly, Perrone et al. [49] found that a great variability was present in the parameters investigated (mycotoxin contamination, altitude, distance from the sea, relative humidity, etc.) among all evaluated grape-producing regions and sometimes within the same region.

In an effort to counter citrus diseases, the development of resistant cultivars through traditional breeding or genetic manipulation appears feasible, since the resistance of citrus to *Alternaria* brown spot is inherited as a recessive trait. Moreover, in the short-term further investigations into the epidemiology, fungicide activity, and application timing might improve disease control.

## Supporting Information

S1 Table The table shows all data used in this paper. The various characters per isolate are reported.
(DOCX)

## References

[1] Logrieco A, Bottalico A, Mulè G, Moretti A, Perrone G. Epidemiology of toxigenic fungi and their associated mycotoxins for some Mediterranean crops. European Journal of Plant Pathology. 2003; 109:645–667.

[2] Akimitsu K, Peever TL, Timmer LW. Molecular, ecological and evolutionary approaches to understanding *Alternaria* diseases of citrus. Molecular Plant Pathology. 2003; 4:435–446. 10.1046/j.1364-3703.2003.00189.x 20569403

[3] Timmer LW, Peever TL, Solel ZVI, Akimitsu K. *Alternaria* diseases of citrus-novel pathosystems. Phytopathologia Mediterranea. 2003; 42:99–112.

[4] Vicent A, Armengol J, Sales R, García-Jiménez J. First Report of *Alternaria* Brown Spot of citrus in Spain. Plant Disease. 2000; 84:1044–. 10.1094/PDIS.2000.84.9.1044B 30832007

[5] Bella C, Guarino R, La Rosa A. Severe infections of *Alternaria* spp. on a mandarin hybrid. Journal of Plant Pathology. 2001; 83:231–.

[6] Elena K. 2006. *Alternaria* Brown Spot of Minneola in Greece; evaluation of citrus species susceptibility. European Journal of Plant Pathology. 2006; 115:259–262.

[7] Peever TL, Ibanez A, Akimitsu K, Timmer LW. Worldwide phylogeography of the citrus brown spot pathogen, *Alternaria alternata*. Phytopathology. 2002; 92:794–802. 10.1094/PHYTO.2002.92.7.794 18943277

[8] Peever TL, Su G, Carpenter-Boggs L, Timmer LW. Molecular systematics of citrus-associated *Alternaria* spp. Mycologia. 2004; 96:119–134. 21148834

[9] Peever TL, Carpenter-Boggs L, Timmer LW, Carris LM, Bhatia A. Citrus Black Rot is caused by phylogenetically distinct lineages of *Alternaria alternata*. Phytopathology. 2005; 95:512–518. 10.1094/PHYTO-95-0512 18943316

[10] Woudenberg JHC, Seidl MF, Groenewald E, de Vries M, Stielow B, Thomma BJ, et al *Alternaria* section *Alternaria*: Species, formae speciales or pathotypes. Studies in Mycology. 2015; 82:1–21. 10.1016/j.simyco.2015.07.001 26951037 PMC4774270

[11] EFSA, 2011. EFSA Journal. 2011; 9 (10):97 Available: http://www.efsa.europa.eu/efsajournal.

[12] Logrieco A, Moretti A, Solfrizzo M. *Alternaria* toxins and plant diseases: an overview of origin, occurrence and risks. World Mycotoxin Journal. 2009; 2:129–140.

[13] Akimitsu K, Tsuge T, Kodama M, Yamamoto M, Otani H. Alternaria host-selective toxins: determinant factors of plant disease. Journal of General Plant Pathology. 2014; 80:109–122.

[14] Howlett BJ. Secondary metabolite toxins and nutrition of plant pathogenic fungi. Current Opinion in Plant Biology. 2006; 9:371–375. 16713733 10.1016/j.pbi.2006.05.004

[15] Markham JE, Hille J. Host-selective toxins as agents of cell death in plant-fungus interactions. Molecular Plant Pathology. 2001; 2:229–239. 10.1046/j.1464-6722.2001.00066.x 20573011

[16] Masunaka A, Tanaka A, Tsuge T, Peever TL, Timmer LW, Yamamoto M, et al Distribution and characterization of AKT homologs in the tangerine pathotype of *Alternaria alternata*. Phytopathology. 2000; 90:762–768. 10.1094/PHYTO.2000.90.7.762 18944496

[17] Ajiro N, Miyamoto Y, Masunaka A, Tsuge T, Yamamoto M, Ohtani K, et al Role of the host-selective ACT-toxin synthesis gene ACTTS2 encoding an enoyl-reductase in pathogenicity of the tangerine pathotype of *Alternaria alternata*. Phytopathology. 2010; 100:120–126. 10.1094/PHYTO-100-2-0120 20055645

[18] Asam S, Rychlik M. Potential health hazards due to the occurrence of the mycotoxin tenuazonic acid in infant food. European Food Research and Technology. 2013; 236:491–497.

[19] Scott PM. Other mycotoxins. Mycotoxins in Food: Detection and Control. 2004; 17:406–440.

[20] Fleck SC, Burkhardt B, Pfeiffer E, Metzler M. *Alternaria* toxins: altertoxin II is a much stronger mutagen and DNA strand breaking mycotoxin than alternariol and its methyl ether in cultured mammalian cells. Toxicology Letters. 2012; 214:27–32. 10.1016/j.toxlet.2012.08.003 22902351

[21] Schwarz C, Tiessen C, Kreutzer M, Stark T, Hofmann T, Marko D. Characterization of a genotoxic impact compound in *Alternaria alternata* infested rice as altertoxin II. Archives of Toxicology. 2012; 86:1911–1925. 10.1007/s00204-012-0958-4 23076116

[22] Saha D, Fetzner R, Burkhardt B, Podlech J, Metzler M, Dang H, et al Identification of a polyketide synthase required for alternariol (AOH) and alternariol-9-methyl ether (AME) formation in *Alternaria alternata*. PloS One. 2012; 7:1–14. 10.1371/journal.pone.0040564 PMC3391263 22792370

[23] Sanzani SM, Reverberi M, Punelli M, Ippolito A, Fanelli C. Study on the role of patulin on pathogenicity and virulence of *Penicillium expansum*. International Journal of Food Microbiology. 2012a; 153:323–331. 22189024 10.1016/j.ijfoodmicro.2011.11.021

[24] Thomas R, 1961. Studies in the biosynthesis of fungal metabolites. Biochemical Journal.1961; 80:234–240. 13776530 10.1042/bj0800234 PMC1243988

[25] Gatenbeck S, Hermodsson S. Enzymic synthesis of the aromatic product alternariol. Acta Chemica Scandinavica. 1965; 19:65–71.

[26] Pryor BM, Michailides TJ. Morphological, pathogenic, and molecular characterization of *Alternaria* isolates associated with *Alternaria* late blight of pistachio. Phytopathology. 2002; 92:406–416. 10.1094/PHYTO.2002.92.4.406 18942954

[27] Simmons EG. *Alternaria*: an identification manual Fully illustrated and with Catalogue Raisonné 1796–2007. CBS biodiversity Centre, Utrecht, The Netherlands 2007.

[28] Sanzani SM, Schena L, De Cicco V, Ippolito A. Early detection of *Botrytis cinerea* latent infections as a tool to improve postharvest quality of table grapes. Postharvest Biology and Technology. 2012; 68:64–71.

[29] White TJ, Bruns T, Lee S, Taylor JW. Amplification and direct sequencing of fungal ribosomal RNA genes for phylogenetics. PCR protocols: a guide to methods and applications. 1990; 315–322.

[30] Glass NL, Donaldson GC. Development of primer sets designed for use with the PCR to amplify conserved genes from filamentous *Ascomycetes*. Applied and Environmental Microbiology. 1995; 61:1323–1330. 7747954 10.1128/aem.61.4.1323-1330.1995 PMC167388

[31] Isshiki A, Akimitsu K, Nishio K, Tsukamoto M, Yamamoto H. Purification and characterization of an endopolygalacturonase from the rough lemon pathotype of *Alternaria alternata*, the cause of citrus Brown Spot disease. Physiological and Molecular Plant Pathology. 1997; 51:15–16.

[32] Hall BG. Building phylogenetic trees from molecular data with MEGA. Molecular Biology and Evolution. 2013; 30:1229–1235. 10.1093/molbev/mst012 23486614

[33] Rasmussen R. Quantification on the Lightcycler. Rapid Cycle Real Time PCR. 2001; 21–22.

[34] Prelle A, Spadaro D, Garibaldi A, Gullino ML. A new method for detection of five *Alternaria* toxins in food matrices based on LC–APCI-MS. Food Chemistry. 2013; 140:161–167. 10.1016/j.foodchem.2012.12.065 23578628

[35] Andersen B, Dongo A, Pryor BM. Secondary metabolite profiling of *Alternaria dauci*, *A*. *porri*, *A*. *solani* and *A*. *tomatophila*. Mycological Research. 2008; 112:241–250. 10.1016/j.mycres.2007.09.004 18262401

[36] Brun S, Madrid H, Gerrits Van Den Ende B, Andersen B, Marinach-Patrice C, Mazier D, et al Multilocus phylogeny and MALDI-TOF analysis of the plant pathogenic species *Alternaria dauci* and relatives. Fungal Biology. 2013; 117:32–40. 10.1016/j.funbio.2012.11.003 23332831

[37] Pruß S, Fetzner R, Seither K, Herr A, Pfeiffer E, Metzler M, et al Role of the *Alternaria alternata* blue-light receptor lreA (whitecollar 1) in spore formation and secondary metabolism. Applied and Environmental Microbiology. 2014; 80: 2582–2591. 10.1128/AEM.00327-14 24532063 PMC3993185

[38] Andersen B, Smedsgaard J, Jørring I, Skouboe P, Pedersen LH. Real-time PCR quantification of the AM-toxin gene and HPLC quantification of toxigenic metabolites from *Alternaria* species from apples. International Journal of Food Microbiology. 2006; 111:105–111. 16890318 10.1016/j.ijfoodmicro.2006.04.021

[39] Ntasiou P, Myresiotis CK, Konstantinou S, Papadopoulou-Mourkidou E, Karaoglanidis GS. Identification, characterization and mycotoxigenic ability of *Alternaria* spp. causing core rot of apple fruit in Greece. International Journal of Food Microbiology. 2015; 197:22–29. 10.1016/j.ijfoodmicro.2014.12.008 25560914

[40] Siciliano I, Ortu G, Gilardi G, Gullino ML, Garibaldi A. Mycotoxin Production in Liquid Culture and on Plants Infected with *Alternaria* spp. Isolated from Rocket and Cabbage. Toxins. 2015; 7:743–754 10.3390/toxins7030743 25751147 PMC4379522

[41] Huang F, Fu Y, Nie D, Stewart JE, Peever TL, Li H. Identification of a novel phylogenetic lineage of *Alternaria alternata* causing citrus Brown Spot in China. Fungal Biology. 2015; 119:320–330. 10.1016/j.funbio.2014.09.006 25937061

[42] Stewart JE, Timmer LW, Lawrence CB, Pryor BM, Peever TL. Discord between morphological and phylogenetic species boundaries: incomplete lineage sorting and recombination results in fuzzy species boundaries in an asexual fungal pathogen. BMC Evolutionary Biology. 2014; 14:1–14. 24593138 10.1186/1471-2148-14-38 PMC4015827

[43] Kohmoto K, Itoh Y, Shimomura N, Kondoh Y, Otani H, Kodama M, et al Isolation and biological activities of two host-specific toxins from the tangerine pathotype of *Alternaria alternata*. Phytopathology. 1992; 83:495–502.

[44] Tsuge T, Harimoto Y, Akimitsu Y, Ohtani K, Kodama M, Akagi Y, et al Host-selective toxins produced by the plant pathogenic fungus *Alternaria alternata*. FEMS Microbiology Reviews. 2013; 37:44–66. 10.1111/j.1574-6976.2012.00350.x 22846083

[45] Desjardins AE, Hohn TM, McCormick SP. Trichotecene biosynthesis in *Fusarium* species: chemistry, genetics and significance. Microbiology and Molecular Biology Reviews. 1993; 57:595–604. 10.1128/mr.57.3.595-604.1993 PMC372927 8246841

[46] Graf E, Schmidt-Heydt M, Geisen R. HOG MAP kinase regulation of alternariol biosynthesis in *Alternaria alternata* is important for substrate colonization. International Journal of Food Microbiology. 2012; 157:353–359. 10.1016/j.ijfoodmicro.2012.06.004 22726725

[47] Van de Perre E, Deschuyffeleer N, Jacxsens L, Vekeman F, Van Der Hauwaert W, Asam S, et al Screening of moulds and mycotoxins in tomatoes, bell peppers, onions, soft red fruits and derived tomato products. Food Control. 2014; 37:165–170.

[48] Andersen B, Nielsen KF, Pinto VF, Patriarca A. Characterization of *Alternaria* strains from Argentinean blueberry, tomato, walnut and wheat. International Journal of Food Microbiology. 2015; 196:1–10. 10.1016/j.ijfoodmicro.2014.11.029 25498470

[49] Perrone G, De Girolamo A, Y Sarigiannis Y, Haidukowski ME, Visconti A. Occurrence of ochratoxin A, fumonisin B2 and black aspergilli in raisins from Western Greece regions in relation to environmental and geographical factors. Food Additives & Contaminants. 2013; 30:1339–1347. 23767884 10.1080/19440049.2013.796594

