# Supplementary material for: Characterization of Citrus-Associated Alternaria Species in Mediterranean Areas
Source: PLoS One. 2016 Sep 16;11(9):e0163255. doi: 10.1371/journal.pone.0163255 (PMC5026349; doi:10.1371/journal.pone.0163255)
Supplement: S1 Table — The various characters per isolate are reported. (DOCX) [file pone.0163255.s001.docx]

| Isolate | Geographical  origin | Tissue of isolation | Morphotype | Genotype | Clade | Pathogenicity on leaf (mm) | Pathogenicity on fruit (mm) | AOH (µg/L) | AME (µg/L) | TEA (µg/L) | ALT (µg/L) | pksH  (log ng) | pksJ  (log ng) | ACT1 (log ng) | ACT2 (log ng) |
| --- | --- | --- | --- | --- | --- | --- | --- | --- | --- | --- | --- | --- | --- | --- | --- |
| A22 | Spain | Fruit | alternata | alternata | I | 42 | 12 | 41 | 25 | 4076 | 4093 | 1.9 | 2.8 | 2.1 | 1.1 |
| A23 | Spain | Fruit | alternata | alternata | I | 24 | 9 | 178 | 105 | 18559 | 1495 | 1.7 | 2.5 | 5.1 | 0.0 |
| A24 | Spain | Fruit | arborescens | arborescens | IV | 28 | 6 | 105 | 22 | 11029 | 33 | 0.9 | 1.6 | 2.8 | 0.0 |
| A25 | Spain | Fruit | alternata | alternata | I | 22 | 8 | 103 | 219 | 7544 | 1115 | 1.1 | 1.8 | 0.0 | 0.0 |
| A26 | Spain | Fruit | tenuissima | alternata | I | 17 | 7 | 27 | 132 | 1082 | 974 | 0.7 | 1.8 | 2.8 | 0.0 |
| A27 | Spain | Leaf | alternata | alternata | I | 22 | 7 | 94 | 104 | 8979 | 351 | 0.9 | 1.5 | 0.0 | 0.0 |
| A28 | Spain | Leaf | alternata | alternata | I | 28 | 14 | 61 | 261 | 4680 | 162 | 2.3 | 3.4 | 2.7 | 1.3 |
| A29 | Spain | Leaf | alternata | alternata | I | 35 | 10 | 24 | 134 | 4181 | 979 | 2.3 | 3.2 | 3.3 | 2.8 |
| A30 | Spain | Leaf | tenuissima | alternata | I | 38 | 11 | 51 | 45 | 4060 | 615 | 2.6 | 3.5 | 4.2 | 3.1 |
| A31 | Spain | Leaf | alternata | alternata | I | 17 | 11 | 31 | 47 | 5729 | 99 | 2.3 | 3.2 | 0.1 | 0.0 |
| A41 | Italy | Leaf | tenuissima | alternata | I | 23 | 10 | 33 | 37 | 2214 | 602 | 2.0 | 3.1 | 3.9 | 0.0 |
| A42 | Italy | Leaf | limoniasperae | alternata | III | 28 | 6 | 13 | 21 | 9570 | 1881 | 3.5 | 3.4 | 0.0 | 0.0 |
| A43 | Italy | Leaf | arborescens | arborescens | IV | 17 | 8 | 61 | 29 | 62 | 667 | 0.5 | 0.7 | 0.0 | 0.0 |
| A44 | Italy | Leaf | citri | alternata | I | 19 | 14 | 132 | 270 | 9306 | 2145 | 3.0 | 3.1 | 0.0 | 0.0 |
| A45 | Italy | Leaf | alternata | alternata | II | 24 | 10 | 54 | 162 | 1587 | 1701 | 3.6 | 3.5 | 3.1 | 0.0 |
| A63 | Italy | Fruit | alternata | alternata | II | 22 | 10 | 68 | 71 | 6901 | 4240 | 3.5 | 3.4 | 0.0 | 0.0 |
| A64 | Italy | Fruit | alternata | alternata | I | 19 | 4 | 54 | 28 | 4442 | 7886 | 1.5 | 2.6 | 0.0 | 0.0 |
| A65 | Italy | Fruit | alternata | alternata | I | 23 | 17 | 34 | 142 | 10033 | 1674 | 2.5 | 3.5 | 0.0 | 0.0 |
| A66 | Italy | Fruit | toxicogenica | alternata | III | 27 | 7 | 46 | 114 | 8613 | 1968 | 2.2 | 3.2 | 0.0 | 0.0 |
| A67 | Italy | Fruit | alternata | alternata | II | 29 | 7 | 43 | 186 | 11635 | 6790 | 2.7 | 3.4 | 0.0 | 0.0 |
